# Extraordinary exposed in early motherhood - a qualitative study exploring experiences of mothers with type 1 diabetes

**DOI:** 10.1186/1472-6874-11-10

**Published:** 2011-04-07

**Authors:** Carina Sparud-Lundin, Marie Berg

**Affiliations:** 1 Institute of Health and Care Sciences, The Sahlgrenska Academy at University of Gothenburg Box 457, SE-405 30, Göteborg, Sweden

## Abstract

**Background:**

Women with type 1 diabetes face several challenges during pregnancy, childbirth and in relation to breastfeeding. It is therefore of utmost importance to consider their need for specific support, early postpartum as well as in daily life after discharge from maternity care. Few studies have investigated these aspects of healthcare. The aim of this study was to explore experiences after childbirth regarding breastfeeding, glycemic control, support and well-being in women with type 1 diabetes.

**Methods:**

A hermeneutic reflective life world research approach was used in this qualitative study. Data was gathered through audio-recorded focus group discussions and individual interviews with 23 women with type 1 diabetes, 6-24 months after childbirth. After verbatim transcription, the text was analyzed in order to identify themes of meaning and a conclusive interpretation of the explored phenomenon.

**Results:**

Experiences of extraordinary exposure challenged the women with type 1 diabetes in their transition to early motherhood. The exposure included a struggle with breastfeeding, although with a driving force to succeed. Everyday life was filled with uncertainty and unpredictability related to one's own unstable glycemic control and the women down-prioritized their own needs in favor of the child. A feeling of being disconnected from professional care further contributed to the experiences of extraordinary exposure.

**Conclusion:**

In early motherhood women with type 1 diabetes have a great need for support in managing daily life postpartum, which requires contemporary approaches to overlap insufficient linkage between health care professionals in maternity and child health care, and diabetes care.

## Background

The childbearing period for women with type 1 diabetes is characterized by several challenges. The relationship between good glycemic control and better outcomes is well documented [1-3]. In order to minimize the increased risk of adverse outcome in diabetic pregnancies, careful preparation before pregnancy and medical care throughout pregnancy should aim to achieve optimal glycemic control [4]. However, stringent glycemic control increases the incidence of severe hypoglycemia [5,6]. This considered, it is not surprising that pregnant women with diabetes experience a higher degree of anxiety, worry, pressure and ambivalence compared to those with low-risk pregnancies [7,8]. The increased frequency of episodes of hypoglycemia is often connected with unfamiliar body responses, unpredictability and loss of control in daily life [9,10].

The pregnancy can become overshadowed by diabetes [11], patients hovering between being enslaved by it and mastering daily life [12]. In our previous study we explored women's need for and experiences of professional support during pregnancy and childbirth. The women with type 1 diabetes received and valued increased attention from health care professionals during pregnancy. However, they considered the attention as being directed towards the health of the unborn baby and not their own health. Furthermore, the women expressed increased vulnerability if the professionals' diabetes specific competence and support was insufficient and if the diabetes organization was disconnected, i.e. lacking communication or collaboration between the antenatal care and the regular diabetes clinic [13].

The challenges for women with diabetes continue during childbirth as frequency of obstetric complications increases, with high rates of caesarean section and instrumental vaginal deliveries [2]. Due to complications and morbidity both in mother and newborn, separation of mother and child soon after childbirth is more common. The risk for hypoglycemia in the baby during the first day (s) [14,15] requires supplemental feeding to maintain glycemic balance. Delayed lacto genesis due to low maternal prolactin levels [16] and immature sucking behavior has been found in these newborns [17]. The mother's insulin requirement often rapidly decreases after delivery of the baby. Together with an increased demand for calories related to breastfeeding, this requires careful individual adjustment of insulin doses in order to avoid severe episodes of maternal hypoglycemia [18]. All of these factors contribute to a risk for delayed initiation, and the need for increased efforts to establish breastfeeding. There are conflicting results concerning breastfeeding rates in women with type 1 diabetes. A Danish study [19] found comparable rates in this group 4 months after childbirth compared to the general population. Two considerably larger German studies [20,21] reported less frequent and shorter duration of breastfeeding. However, these studies reported a positive long-term time trend [21]. The role of professionals is discussed, including aspects of support versus pressure related to breastfeeding in women with diabetes [22]. Clinical routines in the Danish study [19] included several supportive actions to facilitate successful breastfeeding in the women with type 1 diabetes which might explain the high level of breastfeeding rates. For women with type 1 diabetes, who face additional obstacles in relation to breastfeeding, it is of utmost importance to consider their need for specific support, early postpartum as well as in daily life after discharge from maternity care. Few studies have investigated these aspects of healthcare. In this study, the aim was to explore experiences after childbirth regarding breastfeeding, glycemic control, support and well-being in women with type 1 diabetes.

## Methods

The research design of this study is described in a previous publication [13]. In brief, a hermeneutic reflective life world research approach was used. Hermeneutic philosophy stresses that interpretation is a key issue for understanding [23]. A hermeneutic reflective life world research approach emphasizes that understanding of human beings' daily life requires knowledge of how they relate to and interact with the world [24]. Furthermore it strives for adopting an open stance that is sensitive and pliable to a studied phenomenon and includes constraining one's own pre-understanding with the aim of grasping and comprehending meanings [25].

### Settings and Participants

Primi- and multiparous Swedish-speaking mothers with type 1 diabetes at least 6 months after delivery were recruited from four maternity units in the western region of Sweden. The hospitals are located in both rural and urban areas and manage about 160000 deliveries annually. After delivery all these women and their newborns were provided care at the maternity ward according to regional guidelines. These guidelines were similar in the four hospitals and included early feeding of the newborn in order to prevent hypoglycemia, every 3-4^th ^hour during the first 2-3 days. All the mothers were encouraged to breastfeed before every feeding session. The child's blood glucose was measured frequently the first 12 hours, and then with expanded intervals depending on occurrence of hypoglycemia.

The selection and description of the population has been provided in detail in a previous publication [13]. In brief, we identified women with type 1 diabetes through the hospitals' birth register. Thirty-two women received written information about the study by mail. They were then contacted by telephone and after additional verbal information invited to participate in a focus group session. Twenty-five mothers agreed to participate in a focus group discussion, six failed to attend due to last-minute reasons of which two later participated in individual interviews. Two more women were interviewed individually due to living remotely or a desire for privacy. In total, six focus group discussions with 19 women (2-5 in each group) and four individual interviews were performed, with a total of 23 women giving informed consent to participate. All women were Swedish-born, about half of the group had completed secondary school and the others had a university degree. Thirteen of the mothers were having their first child (primiparous) and 10 their second or third child (multiparous). Ten mothers had spontaneous vaginal births, 12 had acute caesareans and one an elective caesarean. The age of the mothers varied between 22-37 years with a diabetes history of 4 to 31 years. The hospital stay varied between one and ten days within the group. Seven of the children had received care in a neonatal care unit. The age of the children at the time of interview varied between 6 and 24 months. The range of breastfeeding duration was one to twelve months. One woman did not breastfeed at all. The study was approved by the Regional Ethics Board [13].

### Data Collection

The focus group discussions, lasting 90 to 120 minutes, were conducted in order to create rich data through interaction between the participants [26,27]. The location was at the University or in a room close to the delivery ward at the hospital. Both researchers participated; one leading the discussion (the moderator) and the other taking notes and asking complementary questions (the observer). The individual interviews lasted 25 to 60 minutes and were conducted by one of the authors; three by telephone and one face-to face [13]. The main question related to this study was: Could you describe your experiences of breastfeeding, glycemic control, diabetes care, support and well-being in daily life after childbirth? The postpartum period was defined to include both the mother's hospital stay and daily life up to six months after discharge. Each woman gave her narrative and clarifying questions were posed followed by common discussion of similar and contrasting situations and feelings. All focus group discussions and interviews were audio-recorded and transcribed verbatim.

### Data Analysis

The interpretative analysis of all text was directed towards discovering qualitative meanings of the phenomenon 'experiences after childbirth regarding breastfeeding, glycemic control, support and well-being in women with type 1 diabetes. No predetermined hypotheses, theories or interpretive sources were used and we strived to set aside our pre-understanding as much as possible with the aim of seeing the "otherness" in the data. The whole was understood in terms of details and details in terms of the whole across all generated data. Meaning units were identified and gradually clustered [25], initially by the first author followed by discussion and reconsideration with the second author, leading to modifications and adjustments of meaning units and emerging structure. Early in the reading of the whole text, an exposure emerged as being central to the mothers' descriptions of daily life after childbirth. Exposure here defined as being emotionally and physically vulnerable and thereby subjected to an exposed position. Therefore, in further reading of the text, the following questions were added in the dialogue with the text: What constitutes the exposure and why does it occur? Is the revelation different to childbearing women in general (without diabetes)? How do the women manage this exposure? What might decrease the exposure? Successively, in continuous dialogue between the authors, themes of meaning were recognized and developed by relating them to each other, looking for similarities and differences. At the end of this process seven themes were formulated. In continuous analysis and explanation of these themes, a further explanation and understanding of the extraordinarily exposure was developed. In this context, extraordinary exposure means being exceptionally exposed, e.g. more vulnerable than childbearing women in general. Finally a conclusive interpretation of the explored phenomenon was formulated.

## Results

The mothers' experiences after childbirth regarding breastfeeding, glycemic control, support and well-being is understood as them being extraordinary exposed. The narratives illuminate that, during the postpartum stay, they felt abnormal compared to other mothers, with extraordinary care requirements related to instable blood glucose both for themselves and their newborns who were also in need of early supplementary feeding; especially when suffering from other neonatal complications:

Your stay at the maternity ward is a little different when you've had a baby and have diabetes. I was in a room with a lot of people who kept coming and going, they went home after a couple of hours....I didn't like it at all....because they had to keep waking us up to check our blood sugar and to give our babies extra feeds. (I:4)

What the exposure constitutes and how mothers dealt with it is organized into seven themes of meaning exemplified in quotes and followed by a conclusive interpretation. The quotes are marked as individual interview (I:1-4) or focus group (FG:1-6).

### The Breastfeeding Struggle

Breastfeeding appeared to be a struggle. The mothers were aware of the child's initial need for early feeding in order to avoid hypoglycemia. The supplementary feeding made it harder to get the child to suck properly at the breast nipple, and often the child became "lazy", preferring the easier method of receiving food - by spoon or bottle. Not being able to establish breastfeeding or having an insufficient amount of breast milk could reveal feelings of pressure, guilt, insufficiency and increased stress:

That was the hardest part; she had to keep eating all the time, because her blood sugar was so low... and I was really stressed because she had to eat so much, so I didn't know how to breastfeed. (F:4)

Supplemental feeding was expressed as a worse alternative than breastfeeding. Some mothers did not understand the care providers' motive for advising it. Other mothers expressed frustration or worry in relation to sometimes inflexible maternity care routines. The negative attitude and behaviour of the health care staff during maternity care contributed to the feeling of frustration and could include inflexible routines such as lack of a common structure for breastfeeding support, when the breastfeeding support was "too physical", or as follows:

He was happy and fell asleep until a night nurse came and told me off, asking if I hadn't been told how dangerous it was (to use a plastic nipple shield) and if he wasn't feeding right ...She said, "He won't get as much as he needs, there might be infections, he could completely lose his ability to suck and so on and so forth"...It made me feel just terrible.

Here I thought I was doing just fine and then she made me feel completely useless. (F:4)

Feelings of guilt and insufficiency could remain long after discharge from maternity care, affecting the mothers' well-being. A great part of their life during this period was dedicated to feeding the baby. One mother expressed that she became depressed for several months due to feeding problems. The struggle to breastfeed was also related to frequent episodes of maternal hypoglycemia. One mother expressed she did not believe that the struggle was worth the fight and perceived that she" got her life back" (F:1) when she gave up breastfeeding and began supplemental feeding.

### Driving Force to Breastfeed

Despite the challenges and obstacles experienced related to establishing breastfeeding, a prominent urge to try and persevere was obvious in the mothers' discussions. One reason for this drive was that other women, without diabetes, breastfeed. The self-image of being a "good" mother made it important to be like those healthy mothers and just make it work; as one mother said: "I have to be able to do it too" (F:1).

For some mothers, the breastfeeding struggle decreased after discharge from hospital. Coming home seemed to promote a reliance on their own resources and a release of pressure: "Actually, I was so happy to be home. I thought, Yes, I can handle this, I'll manage. It wasn't that stressful and not... so much pressure" (F:1). For others the struggle to establish satisfactory breastfeeding routines and endurance to breastfeed could continue for several months:

Mother: I gave him extra feeds with formula and he just cried when I tried to breastfeed.

Moderator: So you must have been really persistent. It seems like it was important for you to breastfeed?

Mother: Yes, that's what the nurse at the paediatric care centre said too, she said, "For goodness sake, give it up! You can just give him some formula."

And I said," Yes, but just let me try one more thing and then one more and one more." After a month I gave him an entire feed (from my breasts), just like that. (F:5)

On the other hand, confidence in the ability to breastfeed was another illuminated aspect. This included expressions of gratitude and sometimes astonishment at how well breastfeeding could eventually work out after the initial struggle. Multiparous women with negative experiences from previous breastfeeding described how they felt more prepared and could thereby manage their blood glucose fluctuations more effectively. As one mother expressed "somewhere at two or three months there was going to be some kind of rhythm to my blood sugar levels and my breastfeeding" (F:6).

### Reinforced Vulnerability

Another dimension of the mothers' exposure after childbirth was an increased vulnerability. Extensive bodily changes with unexpected physical reactions were present and implied unpredictability and a sense of insecurity in daily life. With a few exceptions significant blood glucose fluctuations were experienced, from very high to extremely low levels. When the blood glucose became very low one did not dare rely on the body's signs and signals:

I was dead tired... I saw that they were here but didn't notice how low my blood sugar was then.... I woke up after they gave me the shot. (F:4)

Professionals on the maternity ward could contribute to reinforcement of feelings of vulnerability, as when having insufficient knowledge about diabetes, or being unconcerned or ignorant. This could include not paying attention to the mother and her diabetes; having the only focus on the child's needs.

You're both a new mom and you have diabetes too. You're lonely, vulnerable and fragile, just like any new mom. And then you have this disease, so you need another type of, more intense support from someone who knows about both diabetes and being a new mom. (F:2)

Furthermore, vulnerability could be reinforced when being separated from one's child during the early postpartum period. Sometimes postpartum tiredness and complications hindered the mother-child contact. This was particularly evident when the mother was in the intensive care unit.

So I was lying there just thinking about how much I wanted to see my baby but no one wanted to take me up there and they weren't giving my husband any information. (F:3)

Mothers separated due to the child 's need for neonatal care, felt a need to participate in nursing the child; not be considered as "being in the way". However, some mothers experienced that the neonatal staff dictated the terms for their presence and participation:

They actually didn't ask if I wanted to feed my baby myself. They really made a big deal about it, that I wasn't supposed to be there at all with my son between feeds, just when he had to eat. Then I was supposed to offer him my breast first and then feed him with a cup. (F:1)

To be discharged from hospital, responsible for the child and fearing one's own hypoglycemia was another part of the vulnerability. Frequent hypoglycemic episodes were experienced during the breastfeeding period, leading to feelings of an unpredictable and unsafe daily life which decreased energy and could imply feelings of self-pity. The worst case scenario was becoming unconscious when alone with the child and having responsibility for a child.

Mother 1: I was mostly scared of dropping her on the floor. It was when I was lying down, which is why I didn't see the connection at first.... I'm sort of tired. I had her on my outer side near the edge of the couch but somehow I've always managed to keep a hold of her. And she had fallen asleep because she was full and happy. And then her kicking me and crying woke me up. That didn't feel safe at all.

Mother 2: I can be unconscious with my baby beside me when my husband comes home. This time it's only happened once.... when my husband has been home but it happened several times with my first baby. And nobody contacted me to see how I was doing either, because I was at home. So it's been real difficult. (F:4)

### Restraining glycemic instability

The mothers expressed how they had to struggle with restraining their own glycemic instability. It was a challenge to stabilize blood sugar and it could take one to two months until their insulin requirements became stabilized. Various strategies for managing this were described where decreasing the amount of insulin was the most common action. Very few on the other hand had to increase the dose. For some, dosage had to be reduced gradually, for others very dramatically. As an exception, some mothers had not experienced extensive problems with blood sugar fluctuations. This was interpreted as being related to a more deliberate attitude and a choice of living with higher blood glucose levels. A common experience was that the required adjustment of insulin doses was the mother's own responsibility. Some were confident in doing this; others perceived it as being difficult and felt lonely in the decision-making.

It was a major adjustment since you're used to taking so much insulin and then, just from one day to the next, you go back to your old dose. I thought that was really hard and I took the wrong dose a lot. I went back to my old doses...took too much insulin. (F:6)

Although the mothers were used to planning daily life according to the demands of their diabetes, it was a new experience to have to prepare for breastfeeding occasions in order to avoid hypoglycemia. Consequences of not being able to handle the body's reactions when having hypoglycemia could cause some women to simply wander about, instead of getting food or some sweets. Placing sweets and juice in suitable locations was a part of the preparation in order to obtain a rapid sugar supply of glucose during breastfeeding. Another strategy for dealing with hypoglycemia was to consciously strive for higher blood sugar levels, or to monitor blood glucose more frequently before breastfeeding, especially initially. Avoidance of being alone with the child at home was another way of managing the precarious situation of being a mother of a newborn and having diabetes. The hypoglycemia created insecurity and the perception that breastfeeding could be risky.

Yes, I felt very insecure and that's also the main reason I quit, because I was scared, what if I drop him while I'm sitting here? (F:1)

Several mothers felt a strong need for monitoring and controlling own blood glucose in order not to jeopardize their capacity to take care of the child. While it was sometimes necessary to prioritize one's own diabetes related needs, it could be considered unfair to the baby:

Mother 1: It's just like you say, you have to put yourself first. Both times I've been kind of frustrated...I've really cursed this diabetes, that I have to finish eating....it feels unfair that I can't just, like, pick her up and breastfeed her.

Mother 2: Other babies whose moms don't have diabetes have it different. (F:3)

### Down-prioritizing one's own needs

According to the mothers' narratives, the child and breastfeeding had first priority and it could be difficult to find time to prioritize one's own diabetes-related needs. Despite the awareness of less than perfect diabetes management, an overall expression of satisfaction with the healthy baby and successful breastfeeding was expressed. However, the diabetes was described as being like another child to take care of and breastfeeding and diabetes control were more than a full-time job. There was not enough strength or motivation to maintain the strict glucose control practiced during pregnancy afterwards when the child was "outside their body". A need for relaxation also appeared:

You've been so obsessed with your blood sugar levels during those nine months, so when the baby is born you just think, oh, I'll just check my sugar for the sake of checking but I don't really care what the darn meter says....and then it takes a while before you can take it in, what does the little display really say? So you don't really give a damn about it. (F:5)

### Disconnectedness

Another aspect of the extraordinary exposure evolving from the women's descriptions after discharge from the maternity ward was a sense of disconnectedness and sometimes a feeling of being abandoned. A few had met their diabetes midwife/diabetologist during postpartum care and some had been to a follow-up meeting at the antenatal clinic after discharge. For most of the mothers, support from professionals was experienced as being suddenly interrupted already during care at the maternity ward.

Mother 1: And then, when you've given birth, they suddenly abandon you. No follow-up of how your diabetes is doing. You're supposed to have the time to check your blood sugar when you can't even go to the bathroom. I mean, it's ...nobody gives you any support at all, that's what I felt.

Mother 2: The baby often becomes the centre of attention. You feel like they've forgotten you. I had to get what I needed myself. I wasn't happy when my husband was going back to work. I was worried, what's going to happen if my levels go down really low and what happens if I don't feel well? (I:3)

It had taken several months, sometimes as long as six, before re-establishing the connection with the regular diabetes clinic. The mothers were often expected to make contact themselves, which they did not have the energy to prioritize. When finally visiting the diabetes clinic some diabetes professionals did not pay attention to the passage into motherhood. In addition, with one exception, the child health care professionals seemed to have no knowledge about diabetes in mothers of newborns.

### Adjusting to or questioning available support

Another aspect, contributing to the extraordinary exposure was the fact that contradictory advice in relation to breastfeeding was a frequent issue. The mothers asked for a more coherent structure for communication of individual breastfeeding support, as well as between professionals in different settings such as maternity and neonatal care wards. Being confident in the management of one's diabetes gave courage to take initiative or to express disapproval with the care provided. This seemed to be supported by their experience of mainly intensive, continuous and professional support received during pregnancy. Insufficient professionalism, unreliable follow-up etc. was no longer accepted. For reasons such as these, some mothers had changed or tried to change diabetes care provider. One mother had criticized the diabetes nurse for giving insufficient information about care during the postpartum period. Disappointment could also lower the mothers' expectations and make them try on their own:

And you notice real quickly, whether it's at the delivery ward or in some other health care situation, when they give you that kind of answer. Then you give up, you don't ask any more questions, you work it out yourself. (F:3)

On the other hand, when supportive care providers and problem solving advice was available, it was appreciated:

The midwives at the maternity ward are wonderful, because I had no problem with them kind of grabbing hold of my breast and showing me. I thought their take-charge attitude was great, because I wanted some real help. (F:5)

The home setting with proximity to supportive persons seemed to promote security and the mothers' reliance on her own resources. Support from the child's father/partner (who had been forced to largely adjust to life according to the mother's diabetes) was very decisive for handling daily life with the newborn, the diabetes and breastfeeding issues. Other people could also contribute with important support.

And my husband and I have a routine where we have to talk to each other when he gets to work to make sure I'm out of bed and...I'm sleeping when he goes to work, so he calls and checks that I'm up and he contacts me before he has lunch, during the afternoon and when he's on his way home. And my neighbours know I have diabetes and my oldest daughter who's almost five knows that you can call 112. (F:2)

### Conclusive interpretation: Extraordinary exposed as a mother with type 1 diabetes

The narratives from daily life illuminate that to have diabetes as a mother of a newborn is a situation of extraordinary exposure which seems to challenge the transition to a confident mother. Although the transition to motherhood is an exposed situation for women in general, these mothers have particular needs which make them further exposed. There is a need for extra care of both the newborn and the mother herself related to complications and increased tiredness after a demanding pregnancy and childbirth. To breastfeed is experienced as a pressure and the newborn's initial need of nourishment to avoid dangerous hypoglycemia seem to increase this pressure to breastfeed. A sense of guilt related to the need for using artificial milk is present, which in turn increases the pressure to be a competent mother. Fluctuating glycemic levels, often with unpredictable hypoglycemia are a particular threat to successful breastfeeding, general well-being and the daily routine. The women's own needs are often down-prioritized. The child's health, the family and the maternal role consume a lot of energy, and to feel satisfied with a healthy baby and successful breastfeeding appears to be more important than stabilizing her own blood sugar levels during this period. As a consequence of this extraordinary exposure, the mothers are in need of intensive support; both from professionals and significant others. As one mother described: " The baby's born but you're still really a candidate for maternity care." On the contrary, the intensive professional care and support during pregnancy and childbirth was, for most mothers, abruptly interrupted after the child was born. This led to feelings of being locked out and in "no man's land" in which no professional maternal diabetes support was provided. A sense of loneliness was present and seemed related to the pressure of establishing breastfeeding and stabilizing the often-fluctuating blood glucose levels. Professional support seemed to be almost absent in daily life and taking the initiative to contacting care providers for assistance was not prioritized. The women's partners have to carry a heavy burden of responsibility in the extraordinary exposed situation the family faces. To meet the overall needs of these women awareness of early post partum exhaustion, co-care of mother-child, and maternal involvement in the care of the child is essential. Respite through concrete help, breastfeeding-friendly attitudes and diabetes-related knowledge in care providers are also important.

## Discussion

The findings show that mothers with type 1 diabetes are extraordinary exposed because of the simultaneous challenges of their diabetes and the life-transforming process of motherhood. Motherhood as an exposure has been identified in a theoretical framework developed by Rogan et al. [28]. The process of becoming a mother is characterized by overwhelming change, constant learning and a reconstruction of self, including feelings of being drained, of loss and aloneness. Although we identified a similar process of exposure in mothers with diabetes, it was apparent that their expenditure of physical and emotional energy as well the their need for additional learning was extraordinary. The breastfeeding challenge was extensive; probably more than for newborn mothers in general. Despite the unstable glycemic situation, and other obstacles following childbirth, the desire to breastfeed was obvious and may be related to an awareness of the many benefits for the child. Numerous benefits of breastfeeding for the child have been identified [29]. This is general societal knowledge and might explain the mothers' driving force to breastfeed despite the obstacles they face. This could be a way to balance the guilt for sometimes being forced to take care of their own needs before the baby's. Interconnectedness between breastfeeding and decreased negative mood and perceived stress in mothers has been identified [30,31] and may be even more beneficial for mothers with diabetes. However, women with diabetes undoubtedly need more extensive support from health care providers and relatives to initiate and maintain breastfeeding. There is evidence for the effectiveness of prolonging exclusive breastfeeding by means of additional professional and lay support in promoting any breastfeeding [32]. The question is where such professional support can be provided in times of early postnatal discharge. Barimani et al. [33] discuss the risks related to a lack of linkage in postpartum care, and this became obvious in our study with several mothers experiencing insufficient breastfeeding support, both on the maternity ward and in child healthcare centre.

However, the main finding in this study comprises insufficient and sometimes absent diabetes care to the mothers after childbirth. The extensive support provided to women with diabetes during pregnancies [13] is a widely-spread clinical practice today but a lot remains to be done regarding the health care support after childbirth. Rasmussen et al. [10] have identified the pregnancy and lactation period to be a major turning point and a transition in which women feel that their previous skills in managing their diabetes become inadequate. Our findings indicate that the mothers were more or less left alone with the struggle to manage their diabetes and its unpredictable course after childbirth. In the focus groups, the mothers' need to discuss general issues related to childbirth and breastfeeding was obvious, and compare experiences of how to manage frequent blood sugar fluctuations. Rasmussen et al. [34] have found Internet communication to be one of the main strategies that young women with diabetes in their study used to create stability and manage uncertainty during central transitions such as childbearing. The autonomy and integrity in interactions with others were valued and could thereby control needs for information and support for diabetes-related issues. In line with these findings, one policy could be to provide more or less round-the-clock web-based professional as well as peer support to women in the early motherhood transition.

### Methodological considerations

Combining the strengths of focus groups with those of conducting individual interviews which might contribute with more detailed, deep descriptions can yield different layers of data which complement each other [35]. Life worlds will be encountered and mixed in the focus group discussions, which for the participants, sometimes leads to a new understanding of issues related to the phenomenon [36]. We considered this way of collecting data as a useful tool to capture the meanings of the phenomenon. Furthermore, in line with experiences in another study [37], we found that the focus group discussions allowed the participants to share, reflect on and respond to each others' experiences of meeting everyday challenges. Moreover, the focus group itself became a supportive arena in which feelings and experiences were acknowledged and confirmed by the others, a support the women had needed during pregnancy and after childbirth [13].

One main methodological question is: in what way the findings from this study can be transferred to other practice. Qualitative studies must always be interpreted in relation to the context; in this study, the postpartum period for mothers in Sweden with type 1 diabetes. Studies have shown socio-demographic factors to be predictive of breastfeeding rates in women with diabetes [22]. The participating women in our study had various educational levels, which correspond with Swedish childbearing women in general and were both primi- and multipara. They had experienced different models of antenatal and postpartum care; some had been separated from their child early postpartum while others had not; and their experiences of breastfeeding varied. An important limitation for transferability of the findings was that non-Swedish speaking women were excluded. However, the qualitative study design do not imply that the results will be inapplicable for mothers with type 1 diabetes living in Sweden as well as other countries with differently organized healthcare.

## Conclusion

Entering motherhood after a pregnancy pervaded by extreme attention to blood glucose levels, anxiety about one's own and the child's health, finishing with new challenges after childbirth, should qualify mothers with type 1 diabetes for some kind of directed support during the first months after childbirth. This study shows that the women during their stay at the maternity ward and after discharge from hospital, although extraordinary exposed, did not receive sufficient support. It is obvious that healthcare routines need to be developed, adapted to individual needs and related to both maternal diabetes management and breastfeeding.

## Competing interests

The authors declare that they have no competing interests. Funding was provided by The Swedish Diabetes Association, Capio's Research Foundation and The Baby bag Foundation.

## Authors' contributions

Both authors participated in designing the study; conducting interviews and analysis as well as drafting the manuscript.

## Pre-publication history

The pre-publication history for this paper can be accessed here:

http://www.biomedcentral.com/1472-6874/11/10/prepub
